# Association between Dietary Inflammatory Index and Risk of Colorectal Adenomatous Polyps in Kashgar Prefecture of Xinjiang, China

**DOI:** 10.3390/nu15184067

**Published:** 2023-09-20

**Authors:** Zhuo-Jie He, Weili Yusufu, Shuang Zhang, Min-Yi Luo, Yong-Cheng Chen, Hui Peng, Xing-Yang Wan

**Affiliations:** 1 Department of General Surgery (Anorectal Surgery), The Sixth Affiliated Hospital, Sun Yat-Sen University, Guangzhou 510655, China; hezhj29@mail2.sysu.edu.cn (Z.-J.H.); zhangshuang257@163.com (S.Z.); luomy7@mail2.sysu.edu.cn (M.-Y.L.); 2 Guangdong Provincial Key Laboratory of Colorectal and Pelvic Floor Diseases, The Sixth Affiliated Hospital, Sun Yat-Sen University, Guangzhou 510655, China; chenych76@mail2.sysu.edu.cn; 3 Biomedical Innovation Center, The Sixth Affiliated Hospital, Sun Yat-Sen University, Guangzhou 510655, China; 4 Department of Rectal Surgery, The First Hospital of Kashgar Prefecture, Kashgar 844000, China; valee120@163.com; 5 Department of General Surgery (Endoscopic Surgery), The Sixth Affiliated Hospital, Sun Yat-Sen University, Guangzhou 510655, China

**Keywords:** colorectal adenomatous polyp, dietary inflammation index, diet, inflammation, Kashgar Prefecture

## Abstract

Malignant colorectal tumors and precancerous lesions are closely associated with chronic inflammation. Specific dietary patterns can increase chronic inflammation in the body, thereby promoting the occurrence of tumors and precancerous lesions. We have conducted a case–control study in Kashgar Prefecture, Xinjiang, China, to explore the association between the energy-adjusted dietary inflammatory index (E-DII) and the risk of colorectal adenomatous polyps (CAP). A total of 52 newly diagnosed patients with CAP and 192 controls at the First People’s Hospital of Kashgar Prefecture were enrolled in this study. Dietary information was collected using a food frequency questionnaire. The E-DII was calculated based on dietary data, reflecting an individual’s dietary inflammatory potential. Logistic regression models were used to evaluate the relationship between the E-DII and the risk of CAP, with adjustments for potential confounding factors. The results showed that the maximum anti- and pro-inflammatory values of E-DII were −4.33 and +3.48, respectively. Higher E-DII scores were associated with an increased risk of CAP, and this association remained statistically significant after adjusting for age, sex, body mass index, smoking status, and other relevant variables. Notably, a more pro-inflammatory dietary pattern may be related to an increased risk of developing CAP in Kashgar Prefecture.

## 1. Introduction

Colorectal adenomatous polyps (CAP) are precancerous lesions of colorectal cancer (CRC) that can progress to malignant tumors under certain conditions [1]. The majority of CRC cases arise from polyps, with approximately 70–90% following the adenoma–carcinoma development pathway, and 10–20% developing from serrated lesions [2]. Pathologically, colorectal polyps can be classified into non-adenomatous and colorectal adenomatous polyps (CAP), which are further categorized into tubular, mixed, villous, and serrated adenomas [3,4]. The early detection, diagnosis, and treatment of colorectal malignancies are crucial preventive and therapeutic approaches. Therefore, the early identification and removal of CAP, which form part of an important precancerous state, can effectively prevent progression into malignant colorectal tumors [5,6].

Specific diet and lifestyle habits can result in increased levels of chronic inflammation in the body. For example, the long-term consumption of high levels of red meat and fat or sedentary behavior can increase inflammation, whereas increasing the intake of green vegetables, fruits, and dietary fiber can effectively reduce inflammation levels [7,8,9]. Chronic inflammation plays a significant role in the occurrence and development of colorectal tumors or precancerous lesions through mechanisms such as inducing DNA damage and gene mutations and disrupting the homeostasis of the gut microbiota [10,11,12]. Therefore, assessing the impact of diet on inflammation levels is helpful for formulating appropriate dietary strategies to reduce inflammation. This has significant implications for the prevention and treatment of precancerous lesions and colorectal tumors. The dietary inflammatory index (DII) is used to predict and assess the inflammatory potential of a diet. This can be applied to dietary patterns worldwide. This index was initially proposed by Cavicchia et al. in 2009 and later improved and updated by Shivappa et al. in 2014 [13]. Notably, some researchers have used the energy-adjusted dietary inflammatory index (E-DII) as an alternative to the DII to evaluate the influence of total energy intake. The role of E-DII is consistent with that of DII [14,15]. Although E-DII is not a standard index for inflammation, it suggests the role of dietary risk factors in the development of low-grade chronic systemic inflammation. Previous studies have suggested that low-grade inflammation not only is associated with many chronic diseases, such as hypertension and pathological obesity, but may also potentially establish a mutually reinforcing relationship with tumorigenesis [16]. Recent studies have explored the relationship between DII and the occurrence and development of CRC. In addition, dietary patterns with high levels of inflammation are positively associated with CRC [17,18,19]. Although CAP are an important precursor to CRC, scholars have different views on the association between E-DII and CAP. Consistent with findings from other reports [20], a case–control study conducted in Iran reaffirmed the link between DII and the incidence and development of CRC, while also highlighting a significant correlation with CAP risk, suggesting a potentially pivotal role of DII in early precancerous stages [21]. However, some studies did not find a significant correlation between the DII and CAP. Consequently, more evidence is needed to clarify their relationship further [19,22,23]. Therefore, this study aimed to analyze the correlation between E-DII and the risk of CAP, providing a theoretical basis for conducting dietary intervention guidance and performing early screening for the high-risk population of patients with CAP in Kashgar while offering new perspectives for cancer prevention.

## 2. Materials and Methods

### 2.1. Patients

**Figure 1:**
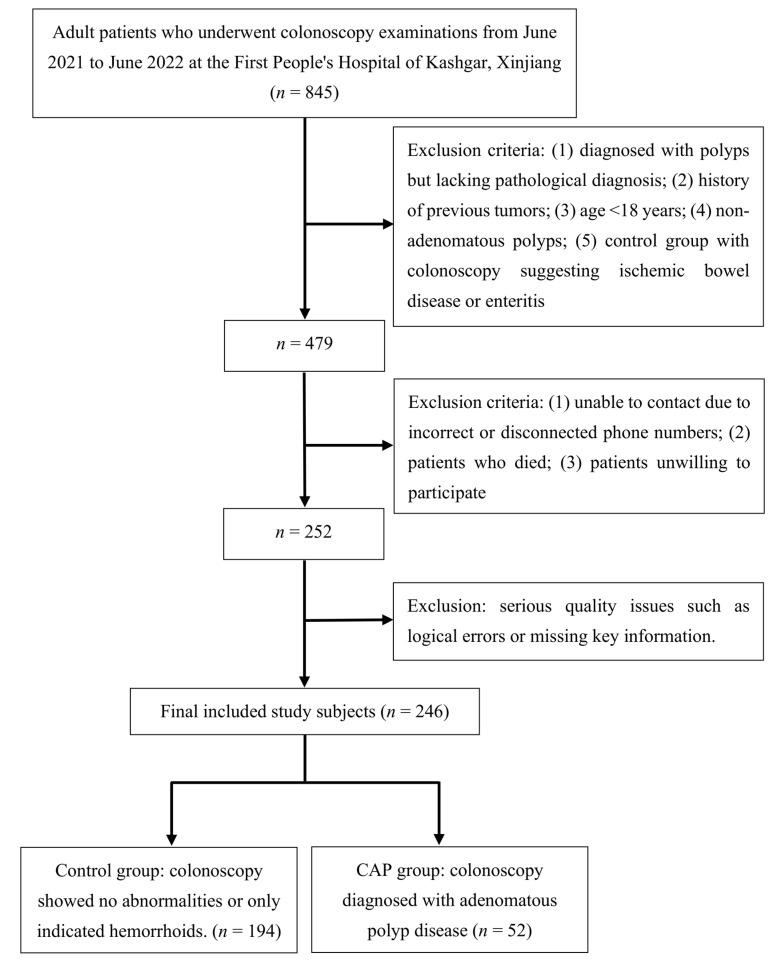
Flowchart of inclusion, exclusion, and grouping in the case–control study conducted in Kashgar, Xinjiang. CAP, colorectal adenomatous polyps.

This study was conducted at First People’s Hospital in the Kashgar region of Xinjiang, China. The case group in this study comprised 52 patients who underwent colonoscopy between June 2020 and June 2021 at the First People’s Hospital of Kashgar and were diagnosed with colonic adenomatous polyps based on pathological examination. The control group comprised 194 patients without a diagnosis of tumors, polyps, or colitis who underwent colonoscopy during the same period in the same hospital’s Department of Digestive Diseases. Six patients were excluded because of poor questionnaire quality. The workflow of this study is illustrated in Figure 1.

### 2.2. Inclusion and Exclusion Criteria

The inclusion criteria were as follows: (1) patients who underwent colonoscopy at the First People’s Hospital of Kashgar between June 2020 and June 2021; (2) no history of colorectal cancer (CRC); (3) for the case group, patients who underwent colonoscopy and were diagnosed with adenomatous polyps based on pathological examination; (4) patients who provided informed consent for this study.

The exclusion criteria were as follows: (1) history of malignant tumors; (2) diagnosis of colitis or ischemic changes in the control group during colonoscopy; (3) diagnosis of CAP without pathological results or non-adenomatous polyps; (4) patients aged < 18 years, pregnant or breastfeeding, suffering from severe mental illness, or unable to care for themselves; (5) patients unwilling to participate, with incorrect or disconnected phone numbers, deceased, or with illegible handwriting, missing responses, or logical errors in the completed questionnaires.

### 2.3. Assessment of Dietary Intake

#### 2.3.1. Data Collection

Trained investigators collected basic clinical and dietary data through telephone interviews. The basic clinical data included sex, age, ethnicity, height, weight, smoking history, alcohol consumption history, medical history, family history, and satiety level. Visual analog scales (VAS) were used to evaluate the satiety levels of the patients after each meal, ranging from 0 to 100%. A rating of 0% indicated “feeling dizzy, nauseous, and weak due to hunger”, whereas a rating of 100% signified “feeling extremely full, unable to consume any additional food”. A rate of 50% denoted “not very full, feel like I can eat a lot more”, and a rating of 80% indicated “feeling somewhat full, with the stomach feeling satisfied” [24,25]. Height and weight measurements were taken without shoes, using a sensitivity of 0.1 cm for height and a mechanical scale with an accuracy of 0.5 kg for weight. The obtained height and weight were used to calculate the body mass index (BMI) using the following formula: BMI = weight (kg)/height (m^2^). According to the World Health Organization classification, we categorized the BMI of the patients into two groups: the obese group, with a BMI ≥ 25 kg/m^2^; and the non-obese group, with a BMI < 25 kg/m^2^ [26]. Dietary data were collected using a semi-quantitative food frequency questionnaire to assess the dietary intake of the patients within the past month. The questionnaire was administered during telephone follow-ups. Recordings were made during the follow-up process, and a random sample of the follow-up questionnaires was reviewed. The dietary questionnaire included 23 types of food, including rice, noodles, meat, fruits, vegetables, and nuts. Participants were asked to recall the dietary frequency of each food item (“never”, “per day”, “per week”, “per month”, or “per year”) and the average intake each time. This information was then converted into daily intake equivalents, and the E-DII score for each patient was calculated based on the nutritional dietary information.

#### 2.3.2. Calculation of DII Score

DII was calculated using the approach reported by Shivappa et al. [13,27]. The food intake data for each patient were converted into nutrient data based on the China Food Composition Table, Standard Edition, Sixth Version. These nutrient values were compared with the global standard nutrient values on the DII scale. Z values were calculated for each nutrient by comparing the patients’ intake with the global database. To mitigate the impact of “right skewing,” the Z values were transformed into a centered percentile score by multiplying the original percentile score by two and subtracting one [(2× percentile of Z value −1)]. This transformation ensures that the values have a symmetric distribution centered around a mean of approximately zero. Based on the specific inflammatory effect score of each nutrient multiplied by the centered percentile score, the individual’s specific DII score for each nutrient was calculated. Finally, the total DII score was obtained by summing all the individual’s specific DII scores for each nutrient. To examine the potential influence of a patient’s total energy intake on the DII, an energy-adjusted version E-DII was used in this study. The calculation of the E-DII was consistent with that of the DII, except that the dietary intake values were converted to per 1000 kilocalorie energy intake values before calculating the Z value. Additionally, standard values and standard deviations from the global database were converted accordingly, with all units expressed per 1000 kilocalorie energy intake [15,28,29]. Referring to previous studies related to the E-DII, prior to the analysis, three quartiles of the E-DII were created based on continuous E-DII scores, which served as the classification criteria for the E-DII as a categorical variable [21,22,27]. Tertiles 1, 2, and 3 represented patients with the lowest, intermediate, and highest potential for dietary inflammation, respectively. To calculate the E-DII score, 27 nutritional parameters, including protein, total fat, carbohydrates, fiber, cholesterol, vitamin A, β-carotene, thiamine, riboflavin, niacin, vitamin C, vitamin E, magnesium, iron, zinc, selenium, folate, saturated fat, monounsaturated fatty acids, polyunsaturated fatty acids, *n*-6 fatty acids, *n*-3 fatty acids, and consumption of onions, garlic, alcohol, and tea, were considered.

### 2.4. Statistical Analysis

We estimated the sample size using the logistic regression module of PASS for Microsoft Windows (ver. 15.0.5; NCSS Inc, Kaysville, UT, USA). Based on the data collected by the research team in the preliminary phase, we anticipate that the incidence rate (P0) of CAP is 20% when E-DII is at the population’s average level, and when E-DII is one standard deviation above the average level, the incidence rate (P1) of CAP is 30%. The correlation coefficient (R²) between E-DII and other confounding variables is set at 0.1 and α is set at a two-sided significance level of 0.05; with the sample size of 246 cases in this study, we expect to achieve a power of 90%. Continuous variables are presented as means with standard deviation (x ± s), whereas categorical variables are presented as percentages (%). The Kolmogorov–Smirnoff test was used to assess the normality of the variable distributions. For normally distributed variables, a *t*-test was used to compare the means between patients and controls, and analysis of variance was used to estimate the means for more than two groups. Nonparametric statistics, such as the Mann–Whitney U test or Kruskal–Wallis test, were used for variables that did not follow a normal distribution. The chi-square test was used to compare the distribution of categorical variables. Logistic regression analysis was used to calculate the odds ratio (OR) with 95% confidence interval (CI) for the risk of colon adenoma formation associated with E-DII. We divided our study population into two groups, the case group (CAP group) and the control group, based on the outcome event. To control for confounding factors, propensity score matching (PSM) and inverse probability of treatment weighting (IPTW) were used to balance and adjust for confounding factors other than E-DII (including age, ethnicity, sex, smoking, diabetes, family history of CRC, mealtime, meat and vegetable pairing, number of breakfasts per week, satiety status per meal, history of Chinese herbal medicine (CHM) use, and BMI). Propensity scores (PS), which integrated various baseline covariates, were calculated for different groups by logistic regression models as the predicted probability of contracting CAP conditional on confounding factors. For PSM, a 1:3 nearest neighbor matching method with a caliper value of 0.05 for the propensity score (PS) was used. For IPTW, patients in the CAP group and the control group were assigned weights of 1/PS (the reciprocal of PS) and 1/(1-PS) (the reciprocal of 1 minus PS), respectively, to achieve balance in multiple confounding factors while maintaining a constant sample size [30,31].

### 2.5. Ethical Approval

This study was approved by the Ethics Committee of the First People’s Hospital of Kashgar Prefecture, and the ethics code of KDYY-EC-SOP-008-03.0.

## 3. Results

### 3.1. Basic Characteristics of the Study Population

This study included 246 participants: 194 and 52 patients in the control and CAP groups, respectively. Using 1:3 PSM, 104 patients in the control group and 42 in the CAP group were selected. The mean of E-DII scores in the present study was −0.29, and the scores ranged from −4.33 to +3.48, where −4.33 and +3.48 represented the most anti- and pro-inflammatory scores, respectively. These results are similar to previous research findings [19,21]. Table 1 presents the descriptive characteristics of the 52 patients with CAP and the 194 controls. When comparing the demographic characteristics and dietary habits between the control and CAP groups, significant differences were observed in terms of sex distribution. The proportion of males was higher in all cases. However, no significant differences were observed between the two groups in terms of other factors, such as age, ethnicity, or smoking history. Table 2 shows the balance of confounding factors before and after applying the PSM and IPTW methods. After matching using the PSM method, the standardized mean differences (SMD) for variables, except for ethnicity, sex, and family history of colorectal tumors, were <0.2, indicating a good balance between the two groups. Similarly, after matching using the IPTW method, the SMD was <0.2 for all covariates, indicating a significant improvement in the balance between the CAP and control groups.

### 3.2. Logistic Analysis of the Relationship between E-DII and the Risk of CAP

Table 3 presents the ORs with 95% CI for the risk of CAP based on E-DII as a continuous variable categorized by tertile. Prior to matching, the results of the multivariable adjusted analysis showed a significant positive association between continuous E-DII and the risk of CAP (OR = 1.30, 95% CI: 1.02–1.67, *p* = 0.035). A similar positive association was observed when analyzing the E-DII based on tertiles. Compared to patients in the first tertile (T1), those in the third tertile (T3) had approximately a threefold higher likelihood of developing CAP (OR = 3.07, 95% CI: 1.23–8.14, *p* = 0.019). After adjusting for confounding factors using the PSM and IPTW methods to reduce their impact, univariate regression analysis was conducted again to evaluate the relationship between E-DII and the risk of CAP. The results showed a consistent positive association, which aligned with the multivariate-adjusted analysis conducted prior to matching.

### 3.3. Subgroup Analysis

**Figure 2:**
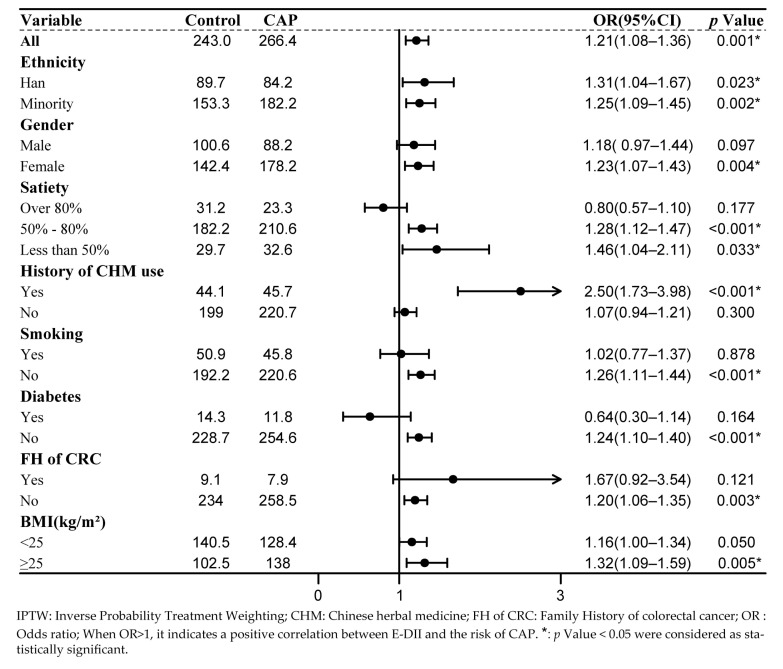
Forest plot of subgroup analysis of selected confounding factor after IPTW.

Subgroup regression analysis was performed for each confounding factor after applying the IPTW method to balance the confounding factors. In the subgroup analysis, a significant correlation between E-DII and the risk of CAP was observed in patients with a history of CHM use. The likelihood of developing CAP in this subgroup increased nearly 2.5 times, whereas no significant correlation was found in the subgroup without CHM use. Similarly, a significant positive association was found between continuous E-DII and the risk of CAP among obese patients (OR = 1.30, 95% CI: 1.02–1.67, *p* = 0.035), whereas no significant association was observed in the non-obese group. Further, a correlation between the E-DII and CAP was observed in the female population but not in the male population. A positive association was found between the E-DII and CAP when the level of satiety was <80%, whereas no significant association was found in the subgroup with satiety levels >80%. The results of the remaining subgroup analyses are shown in Figure 2.

## 4. Discussion

The current study provides evidence that higher E-DII scores in a multi-ethnic population in Kashgar, Xinjiang, China, are associated with an increased risk of CAP, indicating a greater potential for inflammation-related dietary intake. Even after adjusting for known confounding factors through various methods and achieving balanced matching, a significant correlation between the E-DII and CAP risk persisted. However, there is a relative lack of research in this field in Kashgar. Our study contributes to the existing knowledge in this area by providing new evidence supporting the association between pro-inflammatory dietary patterns and the risk of CAP.

Although CAP is recognized as an important precancerous condition of CRC, the relationship between E-DII expression and CAP remains controversial. Our study provides new evidence supporting a positive correlation between CAP risk and E-DII scores. This finding is consistent with previous studies that evaluated the relationship between E-DII scores and the risk of CRC and CAP. Moreover, our study not only confirms previous findings that E-DII is independently associated with CAP, regardless of other risk factors, but also reveals a significant positive correlation between E-DII and CAP specifically in the obese population (BMI ≥ 25 kg/m^2^). In previous studies, adipose tissue has been shown to produce various inflammatory mediators, such as cytokines and hormones. Additionally, obesity can lead to prolonged hypoxia in adipocytes, causing cellular apoptosis, necrosis, and the release of a series of inflammatory mediators. These factors contribute to elevated levels of chronic inflammation in the body, leading to sustained chronic inflammation [32,33]. The strong positive correlation observed between the E-DII and CAP in the obese population may be related to the higher levels of chronic inflammation in this group than in non-obese individuals. Similar results were observed in the subgroup analysis based on the history of CHM use, indicating that certain components of CHM may contribute to a more pronounced relationship between the E-DII and CAP in individuals who use CHM. In China, traditional herbal medicines are extensively used to prevent and treat various diseases. We hope that more extensive and in-depth research will be conducted in the future to further explore this. Our findings revealed a significant correlation between the E-DII and CAP in the obese population and those who had taken herbal medicine. Therefore, targeted early screening and interventions for colorectal cancer in these populations may yield effective preventive outcomes.

In relevant studies, PSM and IPTW methods are considered effective to reduce confounding bias, improve the accuracy of research outcomes, and make them more comparable to randomized controlled trials [34]. PSM achieves group balance through matching treatments; however, the sample size may be reduced due to matching. In contrast, IPTW retains all patient data by increasing the effective sample size through weighting [35,36]. Therefore, by combining the strengths and weaknesses of both methods, this study not only employed a logistic regression analysis method with multifactor adjustment, similar to previous studies, but also conducted regression analysis after balancing the data using PSM and IPTW. The consistent conclusions obtained in this study validate the robustness of the positive association between E-DII and CAP. This further illustrates the correlation between the E-DII and CAP, providing a theoretical basis for the prevention of CAP.

This study had some limitations. Owing to the low rate of colonoscopy screening in Kashgar, Xinjiang, the corresponding screening rates for colorectal tumors and adenomatous polyps are inadequate. This led to a relatively small sample size in the CAP group. As CAP are an important precursor to CRC, there is substantial evidence showing a positive correlation between DII and CRC [17,18,19,20,21]. Further research is needed to explore the association between E-DII and CAP. Prospective studies, such as feeding trials, are necessary. In a recent prospective study, it was also found that E-DII is closely associated with mortality from all causes, including cardiovascular diseases and cancer [28]. This further underscores the significant predictive role of E-DII in many aspects. Due to the limited sample size, we were unable to conduct an in-depth analysis. If this issue is resolved, we could potentially delve more deeply into the association between E-DII and intestinal inflammation, including specific inflammatory markers, which may enhance both the theoretical and practical significance of our study. Additionally, larger sample studies are needed to further analyze the impact of obesity and the previous use of Chinese herbal medicines on CAP. In this study, some unaccounted potential confounding factors, such as sedentary lifestyle and hyperlipemia, could also potentially introduce bias [37,38].

## 5. Conclusions

Our results indicated a significant positive correlation between increasing E-DII scores and the risk of developing CAP. However, owing to the limited sample size in this study, further research with larger sample sizes is needed to validate these findings.

## Figures and Tables

**Table 1:** General characteristics of the 246 study participants included in the research population.

Variables		Control (*n* = 194)	CAP (*n* = 52)	*p*	SMD
Age ^a^	-	49.53(12.76)	52.83(12.74)	0.099	0.259
Ethnicity ^b^	Han	66(34.0)	27(51.9)	0.06	0.369
	Uygur	119(61.3)	23(44.2)		
	Others	9(4.6)	2(3.8)		
Gender ^b^	Male	75(38.7)	30(57.7)	0.021 *	0.388
	Female	119(61.3)	22(42.3)		
Smoking ^b^	Yes	38(19.6)	14(26.9)	0.337	0.174
	No	156(80.4)	38(73.1)		
Diabetes ^b^	Yes	10(5.2)	6(11.5)	0.18	0.232
	No	184(94.8)	46(88.5)		
FH of CRC ^b^	Yes	6(3.1)	4 (7.7)	0.273	0.205
	No	188(96.9)	48(92.3)		
Mealtime ^b^	Regular	166(85.6)	48(92.3)	0.293	0.216
	Irregular	28(14.4)	4(7.7)		
Meat and vegetable pairing ^b^	Mainly vegetarian	40(20.6)	10(19.2)	0.774	0.105
	Mainly carnivorous	7(3.6)	3(5.8)		
	Equally balanced	147(75.8)	39(75.0)		
Number of breakfasts per week ^a^	-	6.48(1.40)	6.69(1.17)	0.316	0.165
Satiety ^b^	Over 80%	24(12.4)	10(19.2)	0.287	0.249
	50–80%	144(74.2)	38(73.1)		
	Below 50%	26(13.4)	4(7.7)		
History of CHM use ^b^	Yes	34(17.5)	11(21.2)	0.69	0.092
	No	160(82.5)	41(78.8)		
BMI ^a^	-	24.40(4.61)	24.95(3.30)	0.421	0.137

^a^: Mean (SD); ^b^: number (percent); SD: standard deviation; SMD: standardized mean difference; FH of CRC: family history of colorectal cancer; CHM: Chinese herbal medicine; BMI: body mass index (kg/m^2^). *: *p* < 0.05 were considered as statistically significant.

**Table 2:** Comparison of variable balance in the matched study population using the propensity score matching and inverse probability of treatment weighting (IPTW) methods.

Variables		PSM				IPTW			
		Control	CAP	*p*	SDM	Control	CAP	*p*	SDM
		*n* = 104	*n* = 42			*n* = 243.03 ^a^	*n* = 266.42 ^a^		
Age ^b^	-	50.57 (11.11)	51.31 (12.20)	0.723	0.064	50.17 (12.70)	51.23 (11.67)	0.615	0.087
Ethnicity ^c^	Han	37 (35.6)	20 (47.6)	0.402	0.246	89.7 (36.9)	84.2 (31.6)	0.722	0.143
	Uygur	61 (58.7)	20 (47.6)			141.9 (58.4)	162.8 (61.1)		
	Others	6 (5.8)	2 (4.8)			11.5 (4.7)	19.3 (7.3)		
Gender ^c^	Male	44 (42.3)	22 (52.4)	0.356	0.203	100.6 (41.4)	88.2 (33.1)	0.298	0.172
	Female	60 (57.7)	20 (47.6)			142.4 (58.6)	178.2 (66.9)		
Smoking ^c^	Yes	27 (26.0)	11 (26.2)	1	0.005	50.9 (20.9)	45.8 (17.2)	0.541	0.095
	No	77 (74.0)	31 (73.8)			192.2 (79.1)	220.6 (82.8)		
Diabetes ^c^	Yes	5 (4.8)	2 (4.8)	1	0.002	14.3 (5.9)	11.8 (4.4)	0.588	0.067
	No	99 (95.2)	40 (95.2)			228.7 (94.1)	254.6 (95.6)		
FH of CRC ^c^	Yes	4 (3.8)	0 (0.0)	0.466	0.283	9.1 (3.7)	7.9 (3.0)	0.733	0.042
	No	100 (96.2)	42 (100.0)			234.0 (96.3)	258.5 (97.0)		
Mealtime ^c^	Regular	92 (88.5)	38 ( 90.5)	0.952	0.066	211.1 (86.9)	227.2 (85.3)	0.841	0.045
	Irregular	12 (11.5)	4 (9.5)			31.9 (13.1)	39.2 (14.7)		
Meat and vegetable pairing ^c^	Mainly vegetarian	19 (18.3)	9 (21.4)	0.838	0.111	49.6 (20.4)	46.3 (17.4)	0.685	0.123
	Mainly carnivorous	4 (3.8)	1 (2.4)			8.5 (3.5)	5.4 (2.0)		
	Equally balanced	81 (77.9)	32 (76.2)			185.0 (76.1)	214.7 (80.6)		
Number of breakfasts per week ^b^	-	6.59 (1.20)	6.62 (1.30)	0.902	0.022	6.52 (1.34)	6.72 (1.15)	0.304	0.156
Satiety ^c^	Over 80%	16 (15.4)	6 (14.3)	0.935	0.068	31.2 (12.8)	23.3 (8.7)	0.708	0.134
	50–80%	79 (76.0)	33 (78.6)			182.2 (75.0)	210.6 (79.0)		
	Below 50%	9 (8.7)	3 (7.1)			29.7 (12.2)	32.6 (12.2)		
History of CHM use ^c^	Yes	21 (20.2)	9 (21.4)	1	0.03	44.1 (18.1)	45.7 (17.2)	0.879	0.026
	No	83 (79.8)	33 (78.6)			199.0 (81.9)	220.7 (82.8)		
BMI ^b^	-	24.70 (4.55)	25.09 (3.54)	0.619	0.096	24.53 (4.56)	24.59 (3.77)	0.942	0.014

^a^: This sample size represents the weighted total population sample size derived from IPTW, which is regarded as a “pseudo-sample size”; ^b^: mean (SD); ^c^: number (percent); SD: standard deviation; SMD: standardized mean difference; FH of CRC: family history of colorectal cancer; CHM: Chinese herbal medicine; BMI: body mass index (kg/m^2^); PSM: propensity score matching; ITPW: inverse probability of treatment weighting.

**Table 3:** Odds ratios and 95% confidence intervals for the association between energy-adjusted dietary inflammatory index and CAP.

Variables	OR	95%CI	*p* Value
Continuous of E-DII ^a^	1.22	1.00–1.51	0.055
Tertiles of E-DII ^a^			
T1	1.00		
T2	1.53	0.68–3.51	0.308
T3	2.27	1.06–5.09	0.039 *
Continuous of E-DII ^b^	1.30	1.02–1.67	0.035 *
Tertiles of E-DII ^b^			
T1	1.00		
T2	2.15	0.84–5.77	0.116
T3	3.07	1.23–8.14	0.019 *
Continuous of E-DII ^c^	1.40	1.09–1.80	0.009 *
Tertiles of E-DII ^c^			
T1	1.00		
T2	2.06	0.74–5.73	0.166
T3	4.05	1.53–10.69	0.005 *
Continuous of E-DII ^d^	1.22	1.08–1.37	0.001 *
Tertiles of E-DII ^d^			
T1	1.00		
T2	2.19	1.38–3.51	0.001 *
T3	2.91	1.84–4.67	<0.001 *

^a^: Univariate logistic regression analysis model; ^b^: multiple factor regression analysis model, adjusted for age, ethnicity, gender, smoking, diabetes, family history of CRC, mealtime, meat and vegetable pairing, number of breakfasts per week, satiety status per meal, history of CHM use and BMI; ^c^: univariate logistic regression analysis model after PSM; ^d^: univariate logistic regression analysis model after IPTW; OR: odds ratio. *: *p* Value < 0.05 were considered as statistically significant.

## Data Availability

The datasets used and/or analyzed during the current study are available from the corresponding author on reasonable request.
